# Lines on Indiginous Medicinal Plants

**Published:** 1866-11

**Authors:** D. L. Phares

**Affiliations:** Newtonia, Miss.


### ARTICLE II.

*Lines on Indiginous Medicinal Plants.* By D. L. Phares, A. M., M. D., of Newtonia, Miss.

*Viburnum Prunifolium.*—This small tree grows in rich, dry woodlands from Florida to the Mississippi river, and northward. For description, see Chapman's "Flora of the Southern United States," and other works. The part used is the bark, ʒ ss to ʒ i, in powder; infusion f ℥ ss; or saturated tincture f ʒ i.

It is nervine, antispasmodic, tonic, astringent, diuretic, and may be used to very good purpose in urinary affections, ophthalmia, aphthous sore mouth, chronic diarrhœa, dysentery, indolent ulcers, etc. It is an excellent remedy in colic, cramp, spasms, palpitation, and other affections incident to pregnancy, or arising from uterine disorder, and for after-pains. But it is *Particularly valuable in preventing abortion and miscarriage, whether habitual or otherwise; whether threatened from accidental cause or criminal drugging.*

It tones up the system, preventing or removing those harassing nervous symptoms that so often torment, wear down, and disqualify the pregnant woman for the parturient effort. It enables the system to resist the deleterious influences of drugs, so often used for the purpose of procuring abortion. It is well known, that the inner bark of the cotton root is used by many to induce miscarriage—one pint of the strong decoction being sufficient for this purpose. The regular exhibition of the viburnum completely neutralizes the effect of the gossypium, compelling the delinquent mother, however unwilling, to carry the fœtus to full term. Some farmers, on whose plantation I have used this medicine, and who have seen much of its effects on negro women who always managed to miscarry, declare their belief, that no woman can possibly abort, if compelled to use the viburnum. This may be claiming too much for it. But it has certainly prevented abortion in every case in which I have ordered it for the purpose. Negatively:—miscarriage has never taken place, so far as I am informed, in any case in which this medicine was used as a preventive.

Brief notes of a few cases will give a better idea of my mode of employing this medicine.

Case I.—Mrs. ——, widely known as authoress, of very pale, delicate appearance, aged about 27, when some three months married, aborted, from injury received in leaping from the floor into bed. Once or twice subsequently, she aborted at the same stage of pregnancy; once, I learned, twins. In August, she came under my care for severe intermittent fever; and, on 16th September, 1864, being again pregnant, she consulted me with a view to prevent abortion. I ordered tincture viburni, f ʒ i bis, *vel ter in die ;* oftener, when threatened, till the danger is passed. She continued going on well for more than three months after the usual time for her misfortunes, when, removing beyond my reach, I lost sight of her. Several times she had to use the medicine very freely. I think it was on the 6th of October, an artillery and cavalry fight took place near the house where she was boarding: her husband, wounded some time before this, was compelled to fly for safety; charges were made through the yard; a number of soldiers were killed about the place; the house was ransacked; and an old gentleman living with the family murdered: yet, she passed safely through this time of excitement and trial.

Case II.—In March, 1865, Mr. W. consulted me in regard to his wife. He said she had never gone to full term, but had had several children at the 8th month, all of them dying one month after birth. Frequent pregnancies and hemorrhages had seriously impaired her health, for improving which I ordered suitable remedies. To prevent premature parturition, she being again pregnant, I directed tincture viburnum. At the 8th month, as usual, labor commenced vigorously, with copious sanguineous discharge. Both were soon arrested by a free exhibition of the viburnum. She went on to full term, and gave birth to a healthy boy, who still survives at a year old.

Case III.—Mrs. M——, mother of several children, has, for several years, suffered much from dysmenorrhœa, leuchorrhœa, hemorrhages, and abortions, and is pale, feeble, and despondent. I ordered iron by hydrogen, to improve the blood and nervous system, Fowler's arsenical solution, to check leucorrhœa and prevent hemorrhage, and tincture viburnum to allay uterine congestion, pain, irritation, and to tone up the reproductive organs. Some months afterwards, March 2d, 1865, I was summoned, in haste, to see her. She was much improved every way, and supposed two or three months pregnant. Two bodies of troops had been ordered to form a junction and prepare for battle, instantly, at a point a mile distant, but visible from the upper story of the dwelling. Running hastily up stairs to see the array, she was hurt: pains commecced, and, almost immediately, pretty free hemorrhage, which alarmed her excessively. A viburnum tree growing in a few paces of the house, I ordered infusion of the bark, which soon put a stop to both hemorrhage and contractions. On the 16th August following, before day, she was alarmed by the escape of liquor amnii, and I saw her early in the morning. As there was no pain, contractions, or other indications of labor, I left her. This was a small leak, and she informed me that labor had been brought on in a previous pregnancy by a similar leak. About dark of the next day, forty hours after the flow commenced, I again saw her, and at 11 P. M. delivered her of a healthy eight months child, which still survives.

Case IV.—January 22d, 1866, Mrs. L——, eighteen months married, had miscarried last year, in consequence of which she had suffered long and much, now pregnant and threatened with abortion. I ordered tincture viburnum f ʒ i thrice a day; oftener if necessary. She went on well till the 10th of April, when she was severely injured by a fall from her carriage. Strong uterine contractions ensued, but were arrested by the medicine, which had to be used freely for several days, gradually diminishing the quantity per diem. For nearly a week abortion was threatened whenever the use of the viburnum was too long omitted. From this time she went on to full term without further accident, and was delivered of a large boy.

Case V.—January 25, 1866, Mrs. H——, married in 1862, has had no children, but an abortion or two, now pregnant, and threatened with abortion at the usual stage with her. I gave her tincture viburnum, with directions to use *pro re nata*. March 4th, summoned again to see her. I find she has had considerable pains, contractions, and discharges for two days. She had taken the medicine as ordered, and was now up, easy, and the discharge a slight oozing merely. Ordered the medicine discontinued for the present. She had to use it again a month later, and from that time continued well, and at full term, gave birth to a healthy child.

Case VI.—July 11th, 1866, Mrs. J——, six or seven months pregnant, has had labor pains increasing in frequency and force for over thirty hours. I ordered tincture viburnum every hour, or as often as needed, till pains cease. Labor was soon arrested; and no further trouble has occurred.

Case VII.—Mrs. P——, April 16, 1866, has had severe colic, after noon, several days. Tincture viburnum f ʒ iss was ordered, and the single dose was all required.

Many cases might be cited, but the above handfull will suffice as well as a thousand. There will be cases and conditions, of course, in which no intelligent practitioner would attempt to prevent abortion by the use of viburnum, or any other means; as where the placenta is extensively detached, the membranes ruptured, the fœtus partly expelled from the uterine cavity, etc.

I have heretofore, for some years past, made known the use of this valuable agent, in conversations with members of the profession, as well as by letter. Its value as a medicine is so well ascertained as to justify a lengthy article in print, and its general use by the medical profession. The bark may be gathered at any time, but is best, perhaps, gathered in October and November. When practicable, I have preferred obtaining it from trees in open, exposed situations. Situation materially affects the qualities of plants. A plant, for instance, which, gathered on the level of New Orleans is inert, gathered here, is probably the best remedy in the world for tetanus: of which, more another time.

